# Sex differences in chemosensation: sensory or emotional?

**DOI:** 10.3389/fnhum.2013.00607

**Published:** 2013-09-26

**Authors:** Kathrin Ohla, Johan N. Lundström

**Affiliations:** ^1^Monell Chemical Senses CenterPhiladelphia, PA, USA; ^2^Division of Psychology, Department of Clinical Neuroscience, Karolinska InstitutetStockholm, Sweden; ^3^Department of Psychology, University of PennsylvaniaPhiladelphia, PA, USA

**Keywords:** sex differences, ERPs, trigeminal, olfactory, GSR, emotion

## Abstract

Although the first sex-dependent differences in chemosensory processing were reported in the scientific literature over 60 years ago, the underlying mechanisms are still unknown. Generally, more pronounced sex-dependent differences are noted with increased task difficulty or with increased levels of intranasal irritation produced by the stimulus. Whether differences between the sexes arise from differences in chemosensory sensitivity of the two intranasal sensory systems involved or from differences in cognitive processing associated with emotional evaluation of the stimulants is still not known. We used simultaneous and complementary measures of electrophysiological (EEG), psychophysiological, and psychological responses to stimuli varying in intranasal irritation and odorousness to investigate whether sex differences in the processing of intranasal irritation are mediated by varying sensitivity of the involved sensory systems or by differences in cognitive and/or emotional evaluation of the irritants. Women perceived all stimulants more irritating and they exhibited larger amplitudes of the late positive deflection of the event-related potential than men. No significant differences in sensory sensitivity, anxiety, and arousal responses could be detected. Our findings suggest that men and women process intranasal irritation differently. Importantly, the differences cannot be explained by variation in sensory sensitivity to irritants, differences in anxiety, or differences in physiological arousal. We propose that women allocate more attention to potentially noxious stimuli than men do, which eventually causes differences in cognitive appraisal and subjective perception.

## Introduction

It is often stated that women have a better sense of smell than men and when sex differences^1^ are reported, women tend to outperform men in odor tasks. However, among the plethora of olfactory sensory studies, differences between men and women almost exclusively exist for tasks that involve odor naming and memory retrieval (Cain, 1982; Doty et al., 1985; Oberg et al., 2002). Importantly, reports of sex differences for detection thresholds, an effective measure of sensory sensitivity, are scarce with a few exceptions originating from studies that used odors with a profound biological or cognitive meaning (Koelega and Koster, 1974; Lundstrom et al., 2003). Inspired by the recent demonstration that women tend to be more reactive to stimuli that are perceived as emotional or irritating (Vigil, 2009), we set out to test the hypothesis that sex differences for chemosensory stimuli are predominantly mediated by differences in cognitive or emotional appraisal rather than sensory sensitivity *per se* by means of both psychological and biometric measures.

In our everyday life, few, if any, odors are processed exclusively by the olfactory system. In most cases, the olfactory and trigeminal systems conjointly process odors. The trigeminal system mediates sensations such as burning, cooling, and tingling, even in the absence of an olfactory percept (Laska et al., 1997). In contrast to what is reported for purely olfactory stimulants, reports of sex-dependent differences in trigeminal sensitivity are more robust in that most studies indeed find significant sex differences. Here, women exhibit higher sensory trigeminal sensitivity (Shusterman et al., 2003), better perceptual acuity (Shusterman and Balmes, 1997; Andersson et al., 2011), and better lateralization ability (Stuck et al., 2006) compared to men. Robust sex differences to purely trigeminal stimulation have been reported in event-related potentials (ERPs) studies (Hummel et al., 1998; Lundstrom et al., 2005; Stuck et al., 2006; Scheibe et al., 2009). These studies reported larger amplitudes and shorter latencies of the late positive component (LPC) of the ERPs to the trigeminal compound carbon-dioxide (CO_2_) for women compared to men (Hummel et al., 1998; Lundstrom et al., 2005). However, although the pronounced sex differences for trigeminal stimuli suggest that women's peripheral trigeminal system is more reactive compared to the sensory system of men, negative mucosa potentials, a non-invasive method to record pain-related electrical potentials from the human respiratory nasal mucosa (Kobal, 1981, 1985), has failed to reveal any sex differences (Frasnelli and Hummel, 2003; Frasnelli et al., 2007). This indicates that the demonstrated sex differences are not primarily mediated by a difference in peripheral processing.

The LPC has been tied to stimulus assessment and evaluation (Polich and Kok, 1995; Pause et al., 1996). Along those lines, sex-dependent differences of the LPC would indicate that women assess nasal irritants differently than men. Further support for the notion of sex differences in stimulus assessment comes from a recent study demonstrating that women tend to be more reactive to stimuli that are perceived as emotional, unpleasant, or threatening (Vigil, 2009), a finding that has been suggested to be indicative of sex-dependent differences in strategies employed when processing emotional stimuli (Hall et al., 2004; Whittle et al., 2011). Women, in general, also exhibit a larger emotional response to sensory stimuli, including intranasal irritation, than men do (Whittle et al., 2011). Importantly, comparative findings exist for the chemical senses. Women report chemical intolerance to a larger degree than men (Johansson et al., 2005; Berg et al., 2008) and women's general responses to intranasal irritation is to a large extent comparable to individuals suffering from chemical intolerance, so-called multiple chemical sensitivity (MCS) (Andersson et al., 2009, 2011), a diagnosis that has been linked to the cognitive processing of the odor rather than sensory acuity *per se* (Hillert et al., 2007). Interestingly, patients with MCS have been successfully treated with a selective serotonin reuptake inhibitor (Andine et al., 1997) the action of which has been linked to a specific reduction of 5-HT1a receptors in the amygdala and insular cortex, both part of the fear processing network (Hillert et al., 2013). Together, these findings suggest that sex-dependent differences for bimodal odors are to some extent linked to the degree of irritation sensation and the cognitive and emotional evaluation of these sensations rather than the sensory processing of the odor alone. In line with that, Ferdenzi et al. (2008) proposed that, starting from young age, women develop a stronger emotional reaction to intranasal sensations than men do. Based on findings from MCS patients, Andersson et al. (2011) recently brought forward the novel hypothesis that a heightened emotional response may render women to allocate more attention toward intranasal stimuli; a potential mechanism mediating previously reported sex-differences.

To assess whether sex-dependent differences in chemosensory processing are primarily mediated by differences in sensory sensitivity or cognitive and emotional appraisal of the stimuli, we used a wide array of measures to capture potential differences in the psychological, sensory, physiological, and neuronal domain. First, we assessed self-reported anxiety before and after stimulation. Furthermore, we measured sensory sensitivity to irritants as well as olfactory discrimination ability. During chemosensory stimulation, ERPs were obtained together with subjective ratings of the stimuli; ERPs provide an ideal tool to distinguish brain processes related to sensory decoding from processes associated with higher cognitive functions, such as attention or memory. At the same time, we measured galvanic skin responses (GSR), a sensitive marker of arousal and emotional responsiveness. As stimulants, we used the mostly odorless CO_2_, which is primarily processed by the trigeminal system with little to no activation of the olfactory system, at high and low irritating concentrations to assess effects of trigeminal irritation independent of odor. In addition, we presented the bimodal odorant cineol at a concentration that combined high irritation and high odorousness.

We hypothesized that sex-dependent differences are mediated by cognitive processes related to attention and stimulus appraisal rather than by differences in sensitivity of the peripheral sensory system. Accordingly, sex-dependent differences should be observed for the LPC. The finding of sex-related differences in measures of subjective anxiety and/or measures of arousal (GSR) would point to differences in emotional responsiveness; an interaction of anxiety and GSR with the LPC effect would suggest that differences in affective processing can modulate cognitive evaluation of the irritants. Conversely to our hypothesis, sex differences for the early ERP components and for thresholds to the irritant would indicate that sex-dependent effects have a predominantly peripheral origin that is in the receptor organ.

## Materials and methods

### Participants

Thirty-seven healthy (no self-reported nasal, psychiatric, and neurological disorders), right-handed participants completed the study. Out of these, eight were excluded before statistical analyses; four due to technical problems during the experiment and four based on excessive movement artifacts that precluded ERP analyses. Consequently, a total of 29 participants, 14 men (25.1 years old, *SD* = 4.8, range = 20–32) and 15 women (25.6 years old, *SD* = 3.8, range = 21–33) were included in all analyses except for GSR. For the GSR analyses, one male participant was excluded due to technical problems with the GSR recording. In order to minimize hormonal influences in the participating women, one third of the women was tested during their follicular phase, during their luteal phase, or while being on hormonal birth control, respectively. Menstrual cycle phase was determined based on retrospective calculation from the point of the onset of the last menses (Lundstrom et al., 2006). Note that effect due to menstrual cycle phase was not assessed due to the limited sample size. All participants were paid for participation and provided written informed consent. The study adhered to the revised Declaration of Helsinki and all aspects of the study were approved by the University of Pennsylvania Institutional Review Board.

### Olfactory identification ability and trigeminal sensitivity

We assessed participants' olfactory identification ability in order to rule out that any of the participants was anosmic by using the Sniffin' Sticks olfactory identification (ID) test (Kobal et al., 1996; Hummel et al., 1997). The test consists of 16 individual felt-tip pens, each containing a distinct odor that is identified using a four-alternative forced-choice paradigm. Two female participants did not participate in the ID test because they were highly familiar with the test and they had achieved high scores previously. Trigeminal sensitivity was assessed for the bimodal odor menthol with a 2-alternative, forced-choice, nostril-laterality detection threshold task using an ascending staircase with 5 reversals (Frasnelli et al., 2011a). For this, 16 concentrations of menthol (R.J. Reynolds Tobacco Company, CAS 2216-51-5, declared purity >99.97%), a bimodal odorant, ranging from 0.1 to 50% with each concentration reduced by one third, were prepared in propylene glycol (1,2 propanediol, Fisher Scientific, Acros Organics, CAS 57-55-6, declared purity >99%) and presented in 60 mL amber glass bottles. Sensory sensitivity to menthol rather than to CO_2_ or cineole, the two stimuli used in the EEG portion of the study, was assessed in order to avoid familiarization to one of the two stimulants. Recent findings suggest that sensitivity to menthol is highly correlated with sensitivity to cineole (Frasnelli et al., 2011a) leading us to believe that thresholds to menthol represent a valid measure of sensitivity to intranasal irritation in a broader sense.

### Subjective anxiety measure

As a measure of subjective anxiety, participants completed the State-Trait-Anxiety Inventory, STAI (Spielberger et al., 1970). The test consists of two parts that assess state and trait anxiety; the range of scores is 20–80 where higher scores indicate greater anxiety. While state anxiety refers to the momentary tendency to experience anxiety, trait anxiety is enduring and universal across different situations (Spielberger and Sydeman, 1994). Both, the STAI-S (state anxiety) and STAI-T (trait anxiety) scores were obtained before and after chemosensory stimulation. STAI-S scores were used to assess possible changes in participants' momentary (state) anxiety attributable to the experimental procedures. Because no such changes were observed, we averaged the pre and post STAI scores (for the S and T subtests) for further analyses.

### Stimuli and procedures

The experiment was conducted in an air-conditioned room constructed specifically for olfactory testing with a high turnover of the total air volume to limit lingering odors. Participants were seated comfortably while EEG was recorded. Chemosensory stimuli were presented using an air-dilution olfactometer (OM6b, Burghart Messtechnik, Wedel, Germany), which embeds the chemosensory stimuli in a continuous stream of humidified (80%) and heated (36°C) air with a flow rate of 6.1 l/min. The methods allows fast rise times of the stimulus (Lorig, 2000) and minimizes somatosensory stimulation from changes in the air flow through the nostrils (Sobel et al., 1998). Stimuli were the non-odorous, trigeminal CO_2_ at 50% v/v and 60% v/v, in the following referred to as CO_2_low and CO_2_high, respectively, and the bimodal, olfactory-trigeminal, cineole (Eucalyptol; Sigma-Aldrich, CAS 470-82-6, declared purity 99%) at 50% v/v. The two concentrations of CO_2_ were selected based on a pilot study (*n* = 5) where the low concentration produced a tactile but no irritating or stinging sensation and the high concentration produced a clear irritating or stinging sensation. All stimuli were presented monorhinally starting with either the right or left nostril and shifting sides halfway at a scheduled break. The olfactometer was placed in a neighboring room to limit acoustic interference and participants were presented brown noise via isolating in-ear headphones to preclude auditory cues from the olfactometer and the shifting air flow.

Each trial started with a central fixation cross presented on a computer screen for a variable interval of 3–9 s. Within this interval, a chemosensory stimulus was presented for 250 ms. Fixation was replaced by the written instruction to rate stimulus irritation, odorousness, and pleasantness on a visual analog scale (VAS) ranging from 1 (not at all) to 100 (extremely strong/pleasant) using the right index finger and a mouse. The rating period started 2.5 s after stimulus delivery. A total of 90 trials (30 trials for each stimulus category: CO_2_low, CO_2_high, and cineole) was presented in pseudo-random order with a variable inter-trial interval of 23.5–38.5 s. Participants were instructed to pay attention to the chemosensory stimulus and to avoid any movements and eye blinks. To allow the presentation of chemosensory stimuli independent of the individual's respiratory cycle, all participants were trained in the velopharyngeal breathing technique, a technique that limits the respiratory flow of air through the nasal cavity (Kobal, 1981), and asked to use this breathing throughout the ERPs portion of the experiment.

### Photo-ionization detection (PID) based timing correction

All mechanical devices exhibit a time-lag between the TTL (transistor-transistor logic) pulse originating from the stimulus computer that initiate stimulus delivery and the actual delivery of the stimulus. This time lag artificially delays the ERP with the corresponding value. We measured the time lag between TTL pulse and arrival of odor molecules at the outlet of the nasal cannula using a fast response miniature photo-ionization detector (PID Mod. 200A, Aurora Scientific inc., Aurora, Ontario, Canada). The sensor has a true frequency response of 330 Hz with a 10–90% rise time of 0.6 ms and the detection limit is 100 ppb (parts per billion) contaminant in air. Onset of the TTL trigger sent and the ongoing PID signal was recorded for 24 continuous stimuli (30 s inter-stimulus interval) per condition using the Powerlab amplifier system (ADInstruments, Colorado Springs, CO) and analyzed using Origin 8.5 (OriginLab, Northampton, MD). Responses were averaged for each condition and latencies from TTL trigger to onset and 50% stimulus concentration—the concentration at which the stimulus approximately starts to be detected—were measured for the three conditions. Averaged measured stimulus onset delays were: cineole 50 ms, CO_2_low 63 ms, and CO_2_high 64 ms. These values were used to temporally adjust the recorded ERP responses to match stimulus onset to the delivery of the stimulant to the receptors rather than to the TTL pulse.

### Galvanic skin responses

Galvanic skin responses (GSR), a non-invasive measure of autonomic nervous system activity [for a comprehensive overview, please see (Stern et al., 2001)], were recorded from bipolar Ag-AgCl electrodes with a surface of 10 mm^3^ according to existing standards (Fowles et al., 1981). The electrodes were placed at the palmar surface of the medial phalanges of the left index and middle fingers. The electrodes were connected to a ML116 GSR amplifier connected to a Powerlab 16/30 system (ADInstruments, Colorado Springs, CO). The amplifier used low constant-voltage AC excitation and automatic zeroing, which reduces electrode polarization artifacts. GSR data were recorded at 200 Hz and analyzed offline using LabChart 7.1 (ADInstruments, Colorado Springs, CO). For analyses, the continuous data were filtered with a 0.01 Hz high-pass filter to remove slow drifts and linear trends. GSR peak amplitudes (in μS) were defined as maximum amplitudes in a 10 s time window after stimulus onset after baseline (500 ms prior to stimulus onset) subtraction.

### Electrophysiological recordings (EEG)

#### Data acquisition and preprocessing

Brain electrical activity was recorded continuously with a BioSemi Active-Two amplifier system (BioSemi, Amsterdam, Netherlands) using 32 Ag/AgCl active electrodes mounted in an elastic cap and placed according to the extended 10–20 system and two additional electrodes, CMS (common mode sense) and DRL (driven right leg) to replace the function of conventional ground electrode (http://www.biosemi.com/faq/cms&drl.htm). Lateral eye movements were monitored with a bipolar outer canthus montage (horizontal electrooculogram). Vertical eye movements and blinks were monitored with a bipolar montage positioned below and above the right eye (vertical electrooculogram). Data were recorded with a sampling rate of 512 Hz and analog filtered from 0.16 to 100 Hz. The continuous EEG signal was stored on a hard disk for off-line analysis.

EEG data were processed using the open-source EEGLAB toolbox (Swartz Center for Computational Neurosciences, La Jolla, CA; http://www.sccn.ucsd.edu/eeglab/; Delorme and Makeig, 2004) running under the Matlab environment (The Mathworks, Inc., Natick, Massachusetts, USA) and the Cartool software by Denis Brunet (brainmapping.unige.ch/cartool). Data were 0.2 Hz high-pass filtered (0.03 Hz transition band width) and segmented into epochs of 3 s (−1000 to 2000 ms relative to stimulus trigger sent to the olfactometer). After manual rejection of epochs with unique, non-stereotypical artifacts, extended infomax independent component analysis (ICA), as implemented in EEGLAB, was applied to the remaining concatenated single trials. Independent components representing common EEG artifacts, such as eye blinks, were visually identified and removed. Back-projected single trials were again screened for residual artifacts. On average, 1% of all trials were rejected leaving an average of 29 trials per condition for further analyses. Data were re-referenced to the averaged mastoids after artifact rejection and correction, and a 30 Hz low-pass filter (1 Hz transition band width) was applied. Subsequently, the onset time of each of the remaining trials was shifted by the stimulus onset delay to the 50% rise latencies obtained from the PID measurement described above. Finally, the baseline (300 ms prior to stimulus onset) was subtracted.

#### Event-related potentials

Event-related potentials (ERPs) were computed for single electrodes before ERPs were averaged across experimental conditions and participants and plotted to visualize the waveform data. Two major ERP deflections were apparent in the grand-averaged ERPs: a minimum (N1) from 200 to 450 ms at centro-lateral electrodes and a slow, positive deflection (LPC) from 400 to 900 ms at centro-parietal electrodes. For statistical analyses, electrodes exhibiting minimum/maximum amplitudes for the N1 and LPC in the grand-averaged waveform were collapsed, a method commonly used to gain statistical power. Then, the minimum peak amplitudes and peak latencies in the 200–450 ms period were extracted at centro-lateral electrodes (FC1, FC2, C3, Cz, C4, Cp1, and Cp2) to characterize the N1. For the LPC, mean instead of peak amplitudes were extracted because the mean amplitude of a slow potential is a more valid measure. For this, mean amplitudes in a 300 ms time window around the peak of the grand-averaged data, i.e., in the 350–650 ms time period for cineole and in the 460–760 ms time period for CO_2_, were extracted at centro-parietal electrodes (FC1, Fz, FC2, Cz, Cp1, Pz, and Cp2).

#### Topographic pattern analyses

It is commonly agreed upon that scalp topographies of the electric field do not change randomly over time, but rather form topographic states that remain stable for periods of several tens of milliseconds; changes in the topography follow from changes in the underlying neural generators (Lehmann et al., 1987). We grouped waveforms into periods of similar topography (also referred to as microstates) using a modified K-means clustering (Pascual-Marqui et al., 1995) as implemented in the Cartool software on the grand-averaged data over the 0 to 1.400 ms interval to identify the predominant maps and their sequence. Model parameters were set such that clusters with a spatial correlation greater than 92% were merged and that each map had to be observed for at least 30 ms. The optimal number of template maps was determined using a combination of criteria: a peak of the modified Krzanowski–Lai criterion (Krzanowski and Lai, 1985) and minimal cross validation. The cluster analysis provides a descriptive means to summarize the ERP data by a limited number of topographic maps.

### Statistical analyses

Statistical analyses were performed with Matlab and SPSS. Initially, we tested for differences between pre- and post-experimental differences in anxiety and submitted the pre-/post-STAI-S and pre-/post-STAI-T scores to Wilcoxon signed-rank tests. This non-parametric test was chosen because the data were not normally distributed. Since pre and post scores were similar for both STAI-S and STAI-T, pre- and post-experimental scores were averaged and used for all further analyses. Then, STAI-S and STAI-T scores and menthol thresholds were submitted to Mann–Whitney *U*-tests in order to test for differences between men and women. Perceptual ratings, GSR, and ERP peak results were submitted to repeated measures ANOVAs with the within subjects factor *stimulant* (CO_2_high, CO_2_low, cineole) and the between subjects factor *sex* (men, women) using SPSS 20.0 (IBM, Armonk, New York, USA). Student's *t*-tests were used for subsequent pairwise comparisons to resolve significant main effects. Huynh-Feld correction for violations of the assumption of sphericity was used when appropriate; uncorrected *F*-values and degrees of freedom and corrected *p*-values are reported. The η^2^ statistic was adopted to describe the estimated proportion of variance explained by the factors. The alpha level was *a priori* set to 0.05.

## Results

### Electrophysiological data (EEG)

**Figure 1:**
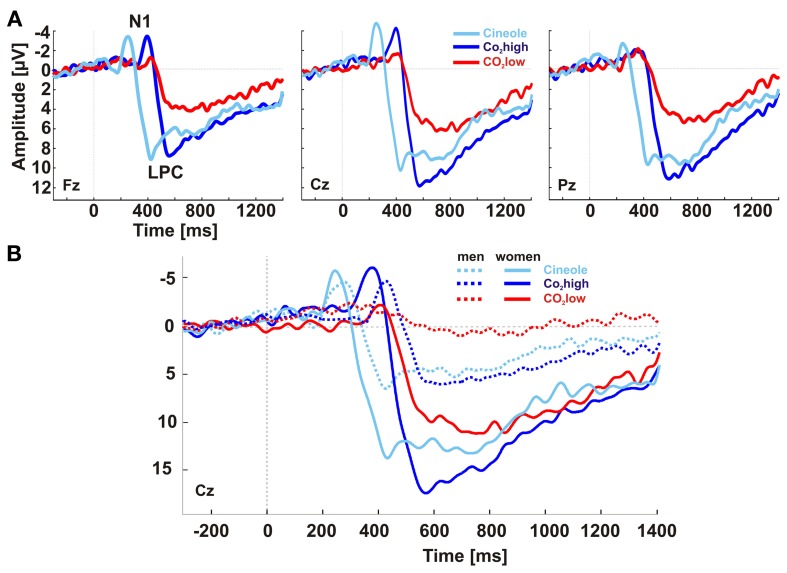
**Baseline-corrected, grand-averaged ERPs at electrodes Fz, Cz, and Pz yielded two major ERP deflections for all stimulants: an early negative peak (N1) and a late slow positive peak (LPC) (A)**. Women exhibited overall shorter latencies and larger amplitudes than men **(B)**.

**Figure 2:**
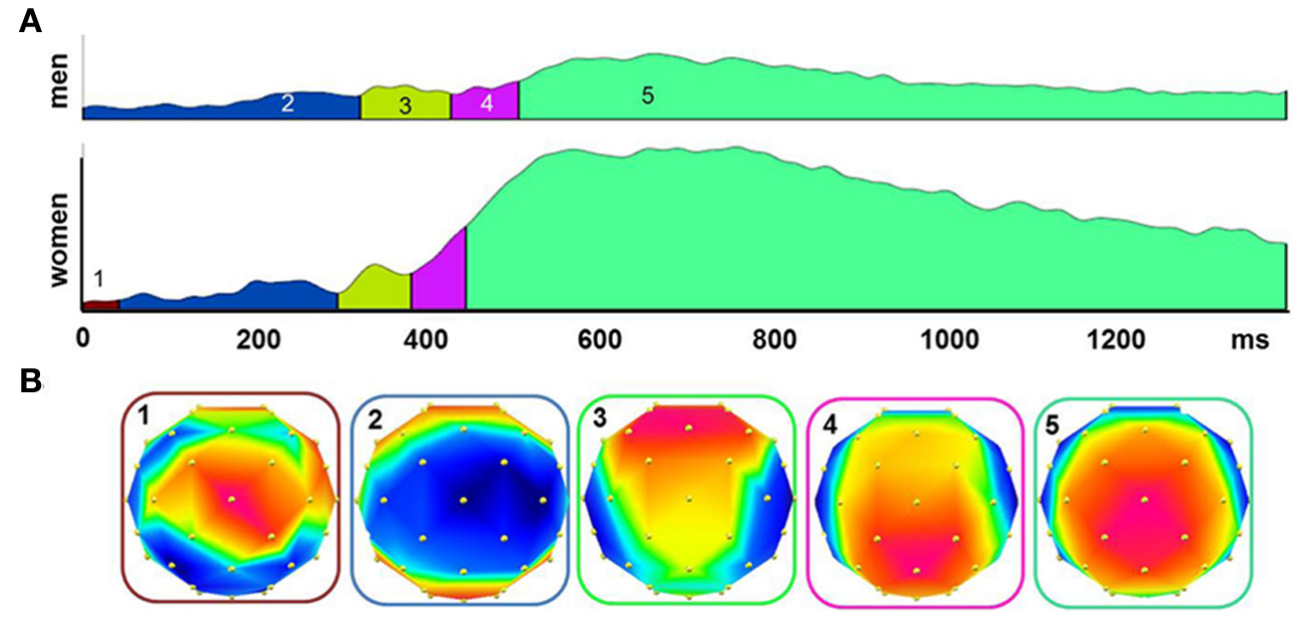
**The global field power (GFP) to all stimulants for men and women were segmented into quasi-stable microstates using a topographic cluster analysis (A)**. Different microstates are indicated by different colors under the curve. Topographical voltage distributions show the signals distribution over the scalp during the period of each microstate **(B)**.

The grand averaged baseline corrected ERPs showed two main deflections at midline electrodes: the N1 with a minimum at around 250 ms for cineole and at 400 ms for CO_2_ over the vertex and adjacent lateral electrodes (i.e., the centro-lateral ROI) and the late positive complex (LPC) with a maximum at around 425 ms for cineole and at 565 ms and 585 ms for CO_2_high and CO_2_low, respectively, over centro-parietal electrodes (Figures 1A, 2B). When comparing men and women, differences in latencies and amplitudes became apparent (Figure 1B, Table 1).

**Table 1:** **ERP peak latencies (in ms) and amplitudes (in μV)**.

		**All**		**Men**		**Women**	
		**Mean**	**SEM**	**Mean**	**SEM**	**Mean**	**SEM**
N1 amplitude	CO_2_low	−0.336	0.042	−0.356	0.0336	−0.317	0.076
	CO_2_high	−0.531	0.065	−0.555	0.100	−0.509	0.087
	Cineole	−0.488	0.050	−0.537	0.054	−0.442	0.081
N1 latency	CO_2_low	334	21.6	325	34.1	342	27.9
	CO_2_high	368	17.2	396	23.2	343	24.1
	Cineole	264	8.4	269	12.2	259	11.9
LPC amplitude	CO_2_low	0.234	0.056	0.018	0.028	0.435	0.072
	CO_2_high	0.530	0.071	0.311	0.058	0.735	0.100
	Cineole	0.504	0.063	0.378	0.088	0.622	0.079
LPC latency	CO_2_low	586	20.5	604	29.6	568	28.7
	CO_2_high	566	15.5	577	22.2	555	22.1
	Cineole	420	12.4	409	18.3	430	16.9

#### Sex-dependent differences in ERP responses

Sex-related ERP differences were found for the amplitude of the LPC only. Women demonstrated higher LPC amplitudes than men as indicated in a main effect of sex [*F*_(1, 27)_ = 19.658, *p* < 0.001, η^2^ = 0.421]. Student's *t-*tests revealed that women exhibited higher LPC amplitudes than men for CO_2_high [*t*_(1, 27)_ = 3.59, *p* = 0.001], CO_2_low [*t*_(1, 27)_ = 5.23, *p* < 0.001], as well as for cineole [*t*_(1, 27)_ = 2.06, *p* = 0.05].

Topographic patterns were subjected to a cluster analysis after the ERPs to the different stimulants were averaged; five microstates explained 95.85% of the variance in the grand-averaged ERP data from 0 to 1400 ms (Figure 2). The topographical voltage maps corresponding to each of the five segments are displayed in Figure 2B. The temporal extent of each map is indicated as colored segments under the global field power (GFP) for each sex. While the sequence of map was highly similar between men and women, the timing was shifted toward faster map occurrence in women suggesting that the underlying cortical generators were similar between the sexes. The first deflection, represented by map 2 (see Figure 2B), with a minimum over centro-temporal sites, constitutes the N1. After a brief transition (map 4), the late positive complex (LPC or P3) established with a maximum over central and parietal electrodes (map 5).

#### Stimulant-dependent effects

**Figure 3:**
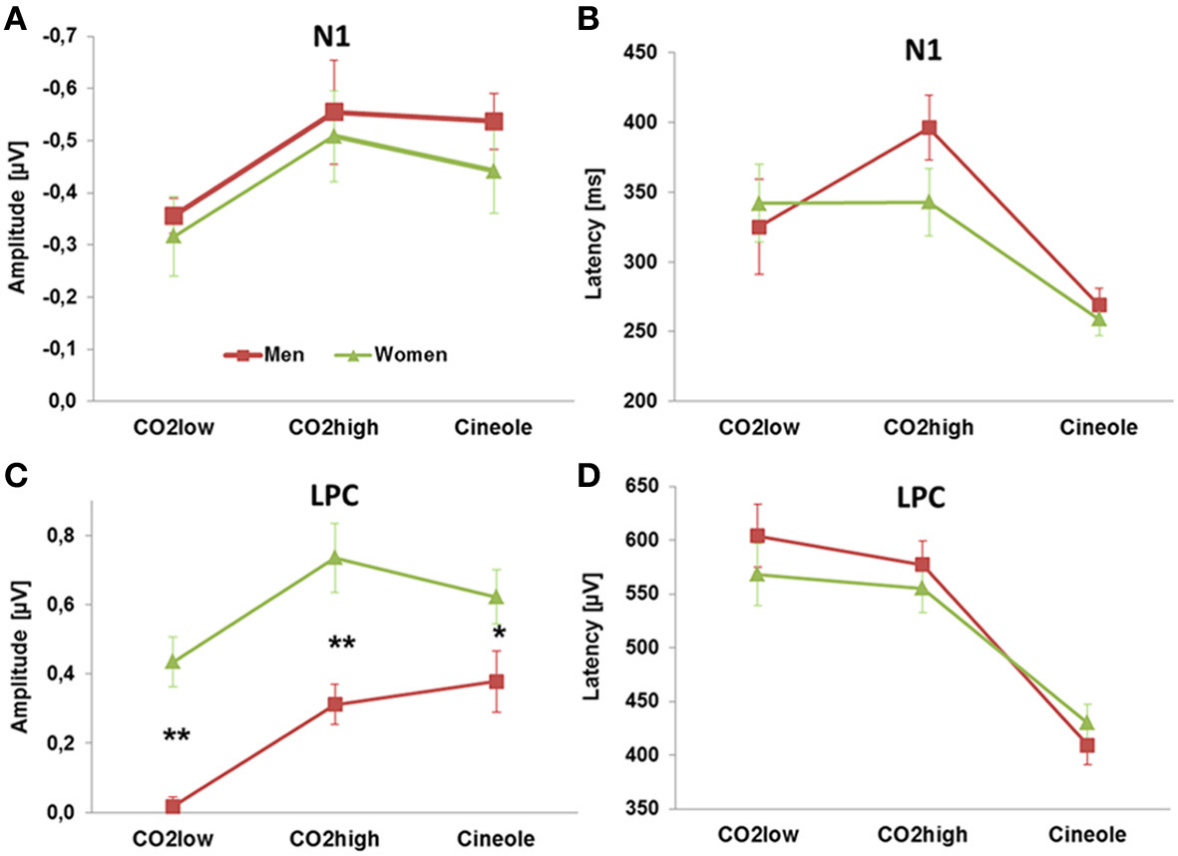
**The amplitudes and latencies of the N1 (A,B) and LPC (C,D) deflections showed effects of stimulants**. Sex-related ERP effects were found only for the amplitude of the LPC **(C)**. ^**^p < 0.001, ^*^p < 0.01.

The ERPs yielded significant differences in response to the three stimulants, as indicated by main effects of stimulant, for N1 peak amplitudes [*F*_(2, 54)_ = 8.87, *p* < 0.001, η^2^ = 0.247], N1 peak latencies [*F*_(2, 54)_ = 17.577, *p* < 0.001, η^2^ = 0.394], LPC peak latencies [*F*_(2, 54)_ = 57.865, *p* < 0.001, η^2^ = 0.682] and LPC mean peak amplitudes [*F*_(2, 54)_ = 15.068, *p* < 0.001, η^2^ = 0.358]. Independent sample paired Student's *t*-tests were subsequently used to resolve the main effects. N1 peaks were most pronounced for CO_2_high and cineole, which were significantly augmented compared to CO_2_low [*t*_(1, 28)_ = 4.591, *p* < 0.001 and *t*_(1, 28)_ = 3.235, *p* < 0.01, respectively]. Similarly, LPC mean peak amplitudes were smaller for CO_2_low than for CO_2_high [*t*_(1, 28)_ = 7.344, *p* < 0.001] as well as for cineole [*t*_(1, 28)_ = 3.895, *p* = 0.001]. N1 latencies were shorter for cineole than for CO_2_low [*t*_(1, 28)_ = 7.866, *p* < 0.001] and than for CO_2_high [*t*_(1, 28)_ = 3.357, *p* < 0.01]. Likewise, LPC latencies were shorter for cineole than both CO_2_low [*t*_(1, 28)_ = 7.876, *p* < 0.001] and for CO_2_high [*t*_(1, 28)_ = 11.378, *p* < 0.001]. Figure 3 displays the amplitudes and latencies of the N1 and LPC for each stimulant and men and women separately.

**Figure 4:**
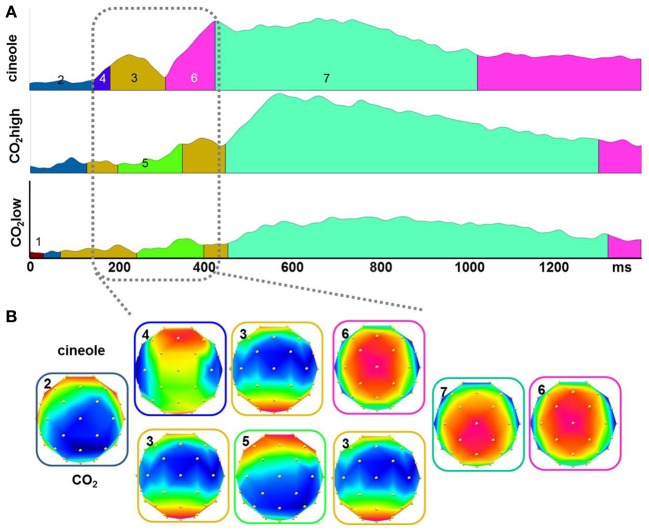
**The global field power (GFP) from all participants for the three stimulants were segmented into quasi-stable microstates using a topographic cluster analysis (A)**. Different microstates are indicated by different colors under the curve. Topographical voltage distributions show the signals distribution over the scalp during the period of each microstate **(B)**. Differences in maps occurrence for the three stimulants were observed during the time period of the N1.

Topographic pattern analyses provided seven microstates accounting for 95.53% of the variance in the grand-averaged ERP data from 0 to 1400 ms of the three experimental conditions. The topographical voltage maps corresponding to each of the seven segments are displayed in Figure 4B. The temporal extent of each map is indicated as colored segments under the GFP for each stimulant (Figure 4A). Differences in map sequence were apparent between cineole and CO_2_ at both intensities; the differences were most prominent during the time period of the N1 deflection and suggest different underlying neuronal generators for the different stimulants.

### Behavioral data

Sensory and behavioral data are summarized in Table 2. All participants scored above 11 on the olfactory identification test (mean = 14.3, SEM ± 0.25, range = 11–16) and we could establish a trigeminal detection sensitivity score in all participants (mean 9.0, SEM ± 0.45, range = 4.8–15.2). There was no significant sex differences in performance for either odor identification (*Z* = 1.48, *p* = 0.138, Mann–Whitney test, 2-tailed) or trigeminal sensitivity (*Z* = 1.004, *p* = 0.315, Mann–Whitney test, 2-tailed). Similarly, anxiety scores were similar in men and women for STAI-S (*Z* = 0.153, *p* = 0.879) and STAI-T (*Z* = 1.638, *p* = 0.101; Mann–Whitney test, 2-tailed).

**Table 2:** **Sensory data, anxiety scores, behavioral ratings, and GSR amplitudes (in μS)**.

		**All**		**Men**		**Women**	
		**Mean**	**SEM**	**Mean**	**SEM**	**Mean**	**SEM**
Odor identification (16-ID)		14.26	0.25	13.86	0.39	14.69	0.29
Menthol threshold		9.00	0.45	8.68	0.68	9.30	0.63
STAI-S^*^		31.84	1.55	32.54	2.75	31.20	1.64
STAI-T^*^		34.05	1.62	36.93	2.80	31.37	1.50
Irritation	CO_2_low	29.78	3.64	23.04	3.97	36.08	5.62
	CO_2_high	56.53	3.97	47.94	6.01	64.55	4.51
	Cineole	70.17	2.12	67.22	3.06	72.92	2.85
Odorousness	CO_2_low	31.48	2.98	30.19	4.08	32.69	4.44
	CO_2_high	38.27	3.16	38.80	3.66	37.78	5.18
	Cineole	59.03	3.37	56.33	4.00	61.56	5.40
Pleasantness	CO_2_low	47.15	1.59	49.29	1.98	45.15	2.39
	CO_2_high	37.08	1.98	39.81	2.50	34.54	2.97
	Cineole	41.93	2.45	38.32	2.34	45.30	4.09
GSR	CO_2_low	0.228	0.04	0.239	0.05	0.216	0.07
	CO_2_high	0.312	0.07	0.382	0.09	0.252	0.10
	Cineole	0.428	0.09	0.525	0.13	0.344	0.13

* Average score before–after.

**Figure 5:**
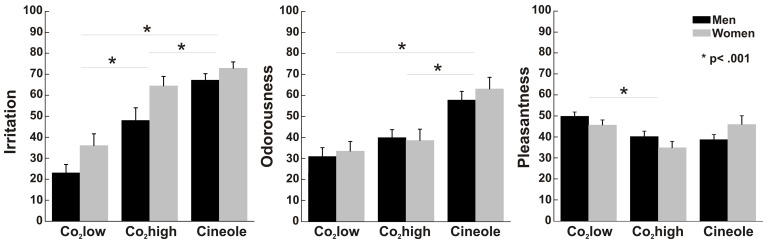
**Perceptual ratings with error bars representing standard error of the mean**. Women rated all stimuli as more irritating than men but no sex differences were found for either pleasantness and or odorousness. In all participants, CO_2_low was least irritating, followed by CO_2_high, and then cineole. Cineole was more odorousness and CO_2_low was most pleasant in all participants.

Figure 5 illustrates participants' ratings to all stimulants. Participants perceived cineole more irritating than CO_2_high [*t*_(1, 28)_ = 3.836, *p* = 0.001] and CO_2_low [*t*_(1, 28)_ = 10.134, *p* < 0.001] and CO_2_high was perceived more irritating than CO_2_low [*t*_(1, 28)_ = 8.671, *p* < 0.001] yielding a main effect of stimulant [*F*_(1, 27)_ = 67.874, *p* < 0.001, η^2^ = 0.715]. A significant sex effect [*F*_(1, 27)_ = 5.757, *p* < 0.05, η^2^ = 0.176] indicated that women rated the stimulants consistently more irritating (mean = 57.8, SEM = 3.36) than men (mean = 46.1, SEM = 3.52).

Perceived odorousness was similar in men and women [*F*_(1, 27)_ = 0.151, *p* = 0.701, η^2^ = 0.006] but it varied for the different stimulants as indicated by a stimulant main effect of Stimulant [*F*_(1, 27)_ = 45.66, *p* < 0.001, η^2^ = 0.628]. As expected, cineole was more odorous than CO_2_low [*t*_(1, 28)_ = 7.343, *p* < 0.001] and CO_2_high [*t*_(1, 28)_ = 6.791, *p* < 0.001]. Surprisingly, the odorless CO_2_high was rated more odorous than CO_2_low [*t*_(1, 28)_ = 4.058, *p* < 0.001] despite both stimulants being considered as odorless in their percept. Difficulties to distinguish between odourousness and intensity/irritation, an observation that some participants reported after the experiment, may have contributed to this finding. We therefore calculated Pearson correlation coefficients between odorousness and irritation ratings. Positive correlations were found between odorousness and irritation ratings for CO_2_low (*r* = 0.62, *p* < 0.001) and CO_2_high (*r* = 0.37, *p* < 0.05) but not for cineole (*r* = 0.29, *p* = 0.119), supporting the notion that participants confused odorousness and irritation for the rather odorless CO_2_high.

Men and women rated the pleasantness of all stimuli similarly [*F*_(1, 27)_ = 0.066, *p* = 0.799, η^2^ = 0.002]. Independent of sex, ratings varied between the three stimulants as indicated by a main effect of stimulant [*F*_(1, 27)_ = 9.191, *p* = 0.001, η^2^ = 0.254]. In detail, CO_2_low was perceived as more pleasant than Co_2_high [*t*_(1, 28)_ = 6.381, *p* < 0.001], which was rated as least pleasant. A significant interaction between stimulant and sex [*F*_(1, 27)_ = 4.244, *p* < 0.05, η^2^ = 0.136] was found. However, pair-wise *t*-tests yielded no significant sex effects for individual odors (all *ts* < 1.5).

### Galvanic skin responses

GSR peak responses were highest for cineole (mean = 0.428, SEM ± 0.09), intermediate for CO_2_high (mean = 0.312, SEM ± 0.07), and smallest for CO_2_low (mean = 0.228, SEM ± 0.04) (see Table 1). Cineole elicited significantly higher GSR than CO_2_high [*t*_(1, 27)_ = 2.373, *p* < 0.05] and CO_2_low [*t*_(1, 27)_ = 3.642, *p* = 0.001] and CO_2_high elicited higher GSR than CO_2_low [*t*_(1, 27)_ = 2.551, *p* < 0.05], resulting in a significant main effect of stimulant [*F*_(2, 52)_ = 9.998, *p* < 0.001, η^2^ = 0.278]. Men and women showed similar responses [*F*_(2, 52)_ < 1]. We subsequently assessed, by means of Pearson's correlation coefficients, whether individual anxiety was reflected in the magnitude of the GSR response. Positive correlations were found between GSR and STAI-T scores in women for all stimulants: CO_2_low (*r* = 0.56, *p* < 0.05), CO_2_high (*r* = 0.62, *p* = 0.01), and cineole (*r* = 0.69, *p* < 0.01). However, no such relation was found in men.

## Discussion

Our results show differential electrophysiological responses to intranasal irritation for women and men: women exhibited significantly increased amplitudes of the late positive ERP potential compared to men. The ERP effect was observed independently of stimulus intensity and of the stimulant used. Yet, women subjectively perceived the stimuli more irritating than men did. Interestingly, men and women were similar with respect to sensory sensitivity, measures of anxiety, and autonomous physiological responses. Consequently, we suggest that women and men process intranasal irritants differently and that this difference is due to cognitive evaluation of the irritants rather than peripheral differences in sensory sensitivity.

We found increased LPC amplitudes along with higher reported irritation in women as compared to men; importantly, the findings occurred in the absence of sex differences in trigeminal sensitivity and anxiety. Augmented ERP amplitudes to trigeminal and bimodal stimuli in women have been described previously for early (Lundstrom and Hummel, 2006) and late potentials (Olofsson and Nordin, 2004; Lundstrom and Hummel, 2006). In most cases, sex-related effect of the ERPs have been interpreted as, or associated with, heightened sensitivity of women compared to men (Olofsson and Nordin, 2004; Stuck et al., 2006). The present data, however, show sex-specific effects to nasal irritation of the LPC only, while the N1, a marker of both exogenous and endogenous stimulus processing (Pause and Krauel, 2000), was similar for both sexes. In contrast to previous findings (Frasnelli et al., 2011b), men and women displayed similar thresholds for menthol, an odorous irritant, and similar odor identification abilities in our study. Taken together, our findings suggest an absence of strong sex-dependent differences in sensory sensitivity toward nasal stimulation.

Chemosensory ERPs have been less investigated in comparison to ERPs derived from the non-chemical senses. It is for that reason that the late positive deflections of the ERP appear to be inconsistently labeled and categorized. Particularly, a clear dissociation between the chemosensory P2 and P3 has yet to be made (Pause, 2002). The LPC in our present study is characterized by a voltage distribution with a parietal maximum which is indicative for a P3 (Polich, 2007). We therefore refer to it as LPC, a P3-like deflection. The LPC has been shown to reflect the cognitive processing of a stimulus (Polich and Kok, 1995; Polich, 2007) including involuntary (re)allocation of attention (Yamaguchi and Knight, 1991), context updating in memory (Donchin and Coles, 1988), and event categorization (Kok, 2001). These processes are achieved after perceptual analyses of the stimulus and comparison of the percept against internal memory representations, leading to the notion that the LPC represents the final step of perceptual processing (Verleger, 1988). Considering the overall cognitive characterization of the LPC, our findings of enhanced LPC amplitudes in women probably reflect differential subjective stimulus evaluation and/or emotional classification. It is prudent to point out that although our data fail to provide evidence for sex-differences in emotional responsiveness to the stimuli, as measured by GSR, it is still possible that differences exist in the emotional classification. It is, however, conceivable that augmented LPC amplitudes in women reflect stronger allocation of attention as a consequence of experience and expectations about the stimuli (Carrion and Bly, 2008). This interpretation is further corroborated by recent findings of Andersson et al. (2011), who demonstrated pronounced sex differences for the LPC with larger amplitudes in women than in men, for both the trigeminal CO_2_ and the bimodal amyl acetate when the stimuli were attended to but not when the stimuli were to be ignored.

Variations in chemosensory perception have been linked to personality (Croy et al., 2011) and individual level of arousal (Pribram and McGuinness, 1975). Based on its intricate connection to the pain system, one of the primary functions of the intranasal trigeminal system is to act as a sentinel that senses irritation from odorous and odorless stimulants and to warn the body against potentially noxious stimuli (Hummel and Livermore, 2002). It is therefore reasonable to assume that increased levels of anxiety and also heightened arousal drive the susceptibility and sensitivity to irritants and that this relation is more pronounced during or directly after the presentation of irritants; especially in comparison to non-irritating odor stimuli. We assessed trait anxiety before testing and state anxiety just before and after nasal stimulation. Men and women showed no significant differences in anxiety scores, a finding that rules out that anxiety in general and, more specifically, anxiety succeeding the stimulation contributed to the observed sex-related differences in chemosensory ratings and the LPC. Notably, we cannot exclude sex-related differences in attitudes toward the stimuli within the present study. Women have reported a higher interest in the sense of smell than men and attitudes were associated with self-reported olfactory sensitivity in a recent study (Seo et al., 2011). However, whether these findings can be readily transferred from olfactory to trigeminal stimulants needs to be demonstrated. Recent findings do suggest, however, that sensitivity to pain represents a distinct category that is independent of sensitivity to odors (Hummel et al., 2011). Furthermore, we measured GSR, a sensitive measure of autonomic arousal that has been shown to be tied to emotional responsiveness and attention (Neumann and Blanton, 1970), during the presentation of the irritants. We found no differences between men and women. The fact that we found no sex-related differences in arousal processing, as measured by GSR and relevant personality traits like anxiety, stronger support our hypothesis that chemosensory sex differences result from higher cognitive processes. Although the exact generators of the LPC have yet to be identified, several cortical, frontal, temporal, parietal, and subcortical limbic, and thalamic structures have been implicated in its generation (Polich, 2007), thus signifying the involvement of a complex neuronal network that may eventually manifest in differential reports of subjectively experienced irritation.

The responses to bimodal and trigeminal stimulation and to different intensities within the trigeminal modality have been described in previous studies (for example, Iannilli et al., 2013). However, it is pertinent to discriminate intensity and/or modality specificity from sex-related ERP differences. In order to address this problem, we presented two different stimulants and two different intensities of the same stimulant. We observed no interaction between stimulants and sex and between intensity and sex indicating that our reported sex differences are independent of the class of stimulants and also of stimulus intensity. Sex-independent differences were, however, observed between stimulants and intensities. When comparing the responses to cineole and CO_2_, we observed an apparent latency shift of the waveform toward faster responses for cineole; this effect was significant for the LPC. Shorter latencies together with higher amplitudes have been reported for CO_2_ in comparison to the non-irritating, less intense phenyl ethyl alcohol (rose-like smell) (Scheibe et al., 2009). Also, different activations pattern of the sensory processing pathways play likely a role: CO_2_ compared to the odorous H_2_S yielded increased activation of the anterior cingulate during the first 140 ms; during the subsequent time period until 320 ms, the orbitofrontal cortex responded stronger to the odor than to the irritant (Iannilli et al., 2013). When comparing CO_2_high and CO_2_low, we observed intensity-dependent shifts of the waveform toward shorter latencies and higher amplitudes for both the N1 and LPC. This observation was expected and is in line with the notion that early deflections of the ERP reflect the processing of sensory properties of a stimulus for non-chemical (Coles and Rugg, 1996) and chemical senses (Ohla et al., 2010). In line with this, Frasnelli et al. (2003) have demonstrated a linear relation between concentrations of CO_2_ and the amplitudes of early and late ERP deflections. Here, the shift of the LPC can be seen as the consequence of the earlier and enhanced perceptual analysis of the stimulus. The latency of the LPC has indeed been shown to be indicative of a difference in the time to detect and evaluate a stimulus (Kutas et al., 1977; Magliero et al., 1984).

In the present study, we used a comprehensive array of psychological, sensory, and psychophysiological measures to investigate sex-related differences in the perception of intranasal irritation. Our results show that women process intranasal irritation differently than men; this effect was manifested in increased irritation perception and enlarged ERP amplitudes of the LPC. Importantly, the differences cannot be explained by variation in sensory sensitivity to irritants or differences in anxiety. We propose that women allocate more attention to potentially noxious stimuli than men do, which eventually causes differences in cognitive appraisal.

### Conflict of interest statement

The authors declare that the research was conducted in the absence of any commercial or financial relationships that could be construed as a potential conflict of interest.

## Footnotes

^1^We are in this report first and foremost interested in effects linked to biological processes rather than gender identity and or societal factors. We are therefore using the term “sex,” a term commonly used when referring to potential differences between men and women based on the underlying biology, rather than “gender,” a term commonly used when referring to sexual (gender) identity.
